# Male Infertility – What about Mental Health?

**DOI:** 10.1055/s-0043-1772471

**Published:** 2023-11-09

**Authors:** William Felipe Fernández-Zapata, Walter Cardona-Maya

**Affiliations:** 1 Department of Microbiology and Parasitology, Universidad de Antioquia, Medellín, Colombia

Dear Editor,

Infertility is the inability to achieve a clinical pregnancy even after engaging in regular and unprotected sexual intercourse for 12 months 1 2 during the fertile days; roughly 8% to 12% of couples of reproductive age worldwide are believed to be affected by infertility. While males are solely responsible for around 20% to 30% of infertility cases, overall, they contribute to half of the cases. 2 It is well known that infertility may induce stress on couples who fail to conceive, but it is unclear whether psychological stress causes infertility. 3 Determining the emotional and psychological impact of male infertility and knowing whether it can improve outcomes if addressed is one of the priorities of male infertility research. 4 Infertile men generally suffer silently, and consequently report lower levels of psychological distress in questionnaires; 5 considering that the concepts of male fertility and virility are part of the perception of masculinity, it is not illogical to assume that there is a social concern that could influence male sexual and reproductive health. 6 A study 7 has found that the pessimism and psychological distress reported by male partners in couples undergoing in vitro fertilization had a negative linear correlation with clinical pregnancy. This might be due to the fact that psychological stress reduces sperm count, progressive motility, and increases abnormal morphology. 8 Another study 9 found an inverse relationship between erectile dysfunction and quality of life measured through the fertility quality of life tool (FertiQoL) and a significant relationship between depression and erectile dysfunction. Additionally, a prospective study 10 suggested infertile men are generally less healthy than their fertile counterparts when considering overall health.

It is unclear whether psychological interventions would be helpful to infertile males; a meta-analysis 11 found a non-significant relationship regarding psychological interventions and depressive symptoms and anxiety in men from infertile couples. However, a systematic review 12 found cognitive behavioral therapy and mind-body interventions proved to be effective psychological interventions, with some positive effects on anxiety, pregnancy rates or marital function, and that coping therapy may be used to reduce stress and anxiety in the waiting period before the pregnancy test.

Male infertility is a clinical challenge; in its guidelines, 13 the European Association of Urology (EAU) recommends that infertile males undergoing fertility therapy should be referred to a mental health professional to undergo different strategies, such as psychoeducation and psychotherapy interventions; nevertheless, the male infertility guidelines 14 of the American Urological Association (AUA) and the American Society for Reproductive Medicine (ASRM) do not include any recommendation related to mental health.

Therefore, male infertility is not only a physical problem; it has emotional and psychological implications. Infertile men can suffer in silence, and psychological stress can negatively affect sperm quality, pregnancy outcomes, and overall quality of life. Although there is conflicting evidence regarding the effectiveness of psychological interventions for infertile men, some studies suggest that cognitive-behavioral therapy and mind-body interventions may be beneficial. However, the current male infertility guidelines differ in their recommendations regarding mental health interventions.

In the Latin American context, male infertility poses significant challenges, as it impacts countless couples due to cultural barriers, limited access to healthcare, socioeconomic disparities, and environmental influences. The ideal conditions for couples trying to conceive involve a supportive and understanding environment that fosters open discussions about infertility. Urgent action and comprehensive strategies are needed to improve sexual and reproductive health outcomes.

To conclude, further research is needed to determine the best approach to treat the psychological impact of male infertility, and healthcare professionals should be aware of the potential mental health implications and consider referral to a mental health professional as appropriate.
